# An asymptomatic mediastinal cyst in a young child: Case report and summary of the literature

**DOI:** 10.1002/ccr3.3070

**Published:** 2020-06-26

**Authors:** Martina Votto, Riccardo Castagnoli, Maria Sole Prevedoni Gorone, Gian Luigi Marseglia, Amelia Licari

**Affiliations:** ^1^ Department of Pediatrics Fondazione IRCCS Policlinico San Matteo University of Pavia Pavia Italy; ^2^ Department of Diagnostic and Interventional Radiology and Neuroradiology IRCCS San Matteo University Hospital Foundation Pavia Italy

**Keywords:** bronchogenic cyst, chest X‐ray, HR‐TC, incidental finding, thoracic surgery

## Abstract

Bronchogenic cyst is a rare congenital chest malformation that mainly presents with wheeze and feeding issues in early life. A multidisciplinary approach and follow‐up are pivotal for the improvement of lung function, mostly in cases of mediastinal complications.

## Graphical abstract

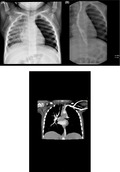

## 1 CASE REPORT

An otherwise healthy 18‐month‐old female child presented to our Pediatric Clinic with a radiological suspect of thoracic mass.

**FIGURE 1:**
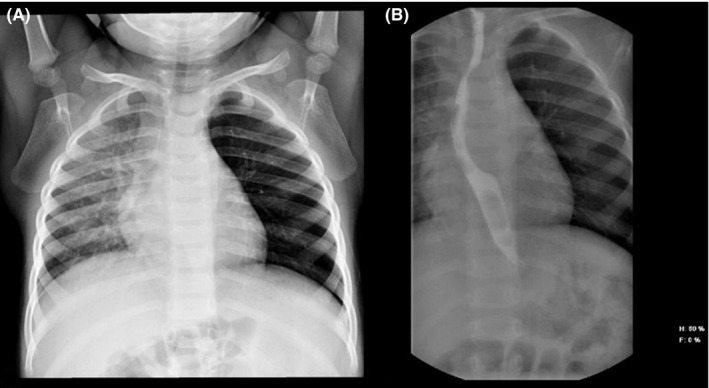
A, Chest X‐ray: diffuse abnormal hyper‐transparency of the left lung fields, with a shift of trachea and mediastinum to the right side, associated with moderate vascular congestion. B, Upper gastrointestinal tract radiography with barium shows an impression on the left side of barium filled esophagus associated with a consequent displacement on to the right side

**FIGURE 2:**
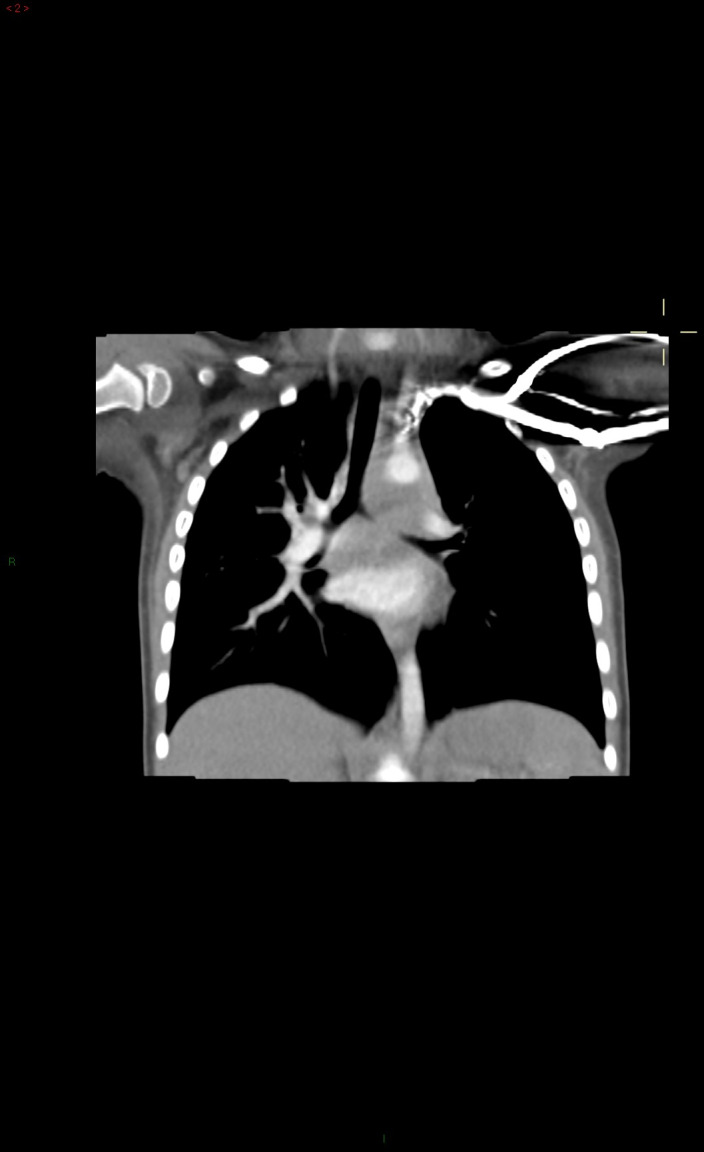
HR‐CT with contrast reveals a large hypodense lesion (34 × 15 × 46 mm) with distinct and smooth margins and fluid density in the posterior mediastinum. This cystic lesion induces the left lung hyperinflation and hypoplasia of left pulmonary artery due to displacement and extrinsic compression of the trachea, left bronchus, and left pulmonary artery

She was born at 40 weeks of gestational age by cesarean section for fetal distress due to prolonged delivery with a wound umbilical cord around the neck. At the moment of birth, she did not need, nor resuscitation nor oxygen therapy and, her birth weight was normal for gestational age. Family history was negative for congenital lung malformations, other lung diseases, and malignancies. No other malformations were reported in the obstetric history. Her past medical history was negative for respiratory distress, recurrent respiratory infections, growth failure, gastroesophageal reflux, and feeding issues. Two weeks before our visit, she developed a mild fever (37.5°C), without any clinical signs of respiratory distress. Thus, she was evaluated by her pediatrician, who requested a chest X‐ray to further investigate the incidental finding of reduced airflow through the left lung, in the suspicion of pneumonia. However, the chest X‐ray—performed in another hospital—revealed a diffuse abnormal hyper‐transparency of the left lung fields, moderate vascular congestion in the right lung, and a shift of trachea and mediastinum to the right side (Figure 1A). Her clinical symptoms disappeared after 2 days without any medications, except the paracetamol, used to manage the fever. At our visit, the child presented good vital signs without respiratory symptoms. We confirmed the reduced airflow through her left lung fields. We evaluated the chest X‐ray with our pediatric radiologists, and we suspected a mediastinal malformation. We performed the echocardiogram that excluded congenital heart defects but revealed hypoplasia of the left branch of the pulmonary artery. Upper gastrointestinal tract radiography with barium showed esophageal compression with consequent dislocation to the right side (Figure 1B). Finally, the child underwent a thoracic high‐resolution computed tomography (HR‐CT with contrast) that revealed a large cystic lesion in the posterior mediastinum (Figure 2) that mainly compressed the left bronchus and the left pulmonary artery. Elective thoracotomy was performed, and the entire thoracic cyst was removed without complications. Histology confirmed the diagnosis of a congenital bronchogenic cyst.

## 2 DISCUSSION

Bronchogenic cysts (BCs) are rare foregut‐derived congenital cystic malformations of the respiratory tract. BCs derived from abnormal budding of the tracheal diverticulum before 16 weeks of gestational age and appear as a closed sac typically made up of respiratory epithelium.^1^ The exact epidemiology is unknown, but it is estimated that it may affect 1/42.000‐1/68:000 people.^2^ BCs are usually located in the mediastinum (85% of cases) and are classified based on their location in (a) paratracheal, (b) carinal, (c) paraesophageal (possible presence of a fistula), and (d) hilar cysts; however, unusual locations (neck, abdomen, or pericardium) are also described.^3^

Bronchogenic cysts can progressively enlarge during fetal growth and overtime after birth. Symptoms can develop at any age and are due to the mass effect and the compression of mediastinal airways, vascular vessels, and the esophagus. The vast majority of affected children present persistent wheeze and cough (70%); however, other symptoms may concern feeding issues (dysphagia and regurgitations) and failure to thrive.^4^ BCs may complicate with infection and may present with chest pain, fever, cough, and acute respiratory distress.^4^ In asymptomatic patients, the diagnosis is incidental and may mainly occur in adulthood.^4^ Thus, initially, silent lesions may be diagnosed in adult patients because of complications or suspected malignancy.^4^ The diagnostic work‐up may include the chest X‐ray, barium swallow, bronchoscopy, and HR‐CT scan or MRI. Thoracic CT scan or MRI may be highly suggestive of a bronchogenic cyst, but the histology is required for a definitive diagnosis, especially in uncertain cases.^5^ BCs might be distinguished from other mediastinal congenital cysts, such as intestinal duplication cyst. Infected and suppurated BCs could appear as a lung abscess.

Bronchogenic cyst is typically a single spherical cyst with a smooth appearance and a thicker wall. In some cases, the diagnosis might be made during pregnancy with ultrasounds or MRI.^4^ The complete surgical excision is the only curative therapy in symptomatic patients.^4^, ^5^ Elective excision is also recommended in asymptomatic cases, preventing future symptoms and complications (compression of mediastinal structures, recurrent infections).^4^, ^5^ Lung function—previously compromised by the BC—generally improved after the surgical excision, and a strict follow‐up is required in children with a history of recurrent respiratory infection and lung hypoplasia.^4^ BCs might be rarely associated with vertebral malformations.^4^, ^5^

In conclusion, BC is an extremely rare congenital malformation of the tracheobronchial tree that may present with persistent or recurrent respiratory symptoms in the first years of life. However, BCs may be rarely asymptomatic, resulting in an incidental finding. We reported a case of BC in an otherwise healthy young child. In asymptomatic cases, clinical suspects may arise from an accurate physical examination and diagnostic work‐up. A multidisciplinary approach (including pediatricians, pulmonologists, gastroenterologists, radiologists, and surgeons) is pivotal for a correct diagnosis and therapeutic success. After surgical excision, a strict follow‐up is needed to evaluate the improvement of lung function, especially in children with pulmonary hypoplasia or previous recurrent respiratory infections.

## CONFLICT OF INTEREST

None declared.

## AUTHORS' CONTRIBUTION

MV, RC, and AL: conceived the idea for the article, contributed to patient management, and prepared the final version of the manuscript. MSP and GLM: reviewed the manuscript critically. All the authors have read and approved the final draft of the manuscript.
